# Spared speech fluency is associated with increased functional connectivity in the speech production network in semantic variant primary progressive aphasia

**DOI:** 10.1093/braincomms/fcad077

**Published:** 2023-03-16

**Authors:** Maxime Montembeault, Zachary A Miller, Amandine Geraudie, Peter Pressman, Antoine Slegers, Carly Millanski, Abigail Licata, Buddhika Ratnasiri, Maria Luisa Mandelli, Maya Henry, Yann Cobigo, Howard J Rosen, Bruce L Miller, Simona M Brambati, Maria Luisa Gorno-Tempini, Giovanni Battistella

**Affiliations:** Memory and Aging Center, Department of Neurology, University of California in San Francisco, San Francisco, CA 94158, USA; Douglas Mental Health University Institute, Montréal, QC H4H 1R3, Canada; Department of Psychiatry, McGill University, Montréal, QC H3A 1A1, Canada; Memory and Aging Center, Department of Neurology, University of California in San Francisco, San Francisco, CA 94158, USA; Memory and Aging Center, Department of Neurology, University of California in San Francisco, San Francisco, CA 94158, USA; Department of Neurology, Toulouse University Hospital, Toulouse 31400, France; Department of Neurology, Behavioral Neurology Section, University of Colorado Anschutz Medical Campus, Aurora, CO 80238, USA; Département de Psychologie, Université de Montréal, Montréal, QC H3C 3J7, Canada; Centre de recherche de l’Institut Universitaire de Gériatrie de Montréal, Montréal, QC H3W 1W5, Canada; Memory and Aging Center, Department of Neurology, University of California in San Francisco, San Francisco, CA 94158, USA; Department of Speech, Language, and Hearing Sciences, The University of Texas at Austin, Austin, TX 78712-0114, USA; Memory and Aging Center, Department of Neurology, University of California in San Francisco, San Francisco, CA 94158, USA; Memory and Aging Center, Department of Neurology, University of California in San Francisco, San Francisco, CA 94158, USA; Memory and Aging Center, Department of Neurology, University of California in San Francisco, San Francisco, CA 94158, USA; Department of Speech, Language, and Hearing Sciences, The University of Texas at Austin, Austin, TX 78712-0114, USA; Memory and Aging Center, Department of Neurology, University of California in San Francisco, San Francisco, CA 94158, USA; Memory and Aging Center, Department of Neurology, University of California in San Francisco, San Francisco, CA 94158, USA; Memory and Aging Center, Department of Neurology, University of California in San Francisco, San Francisco, CA 94158, USA; Département de Psychologie, Université de Montréal, Montréal, QC H3C 3J7, Canada; Centre de recherche de l’Institut Universitaire de Gériatrie de Montréal, Montréal, QC H3W 1W5, Canada; Memory and Aging Center, Department of Neurology, University of California in San Francisco, San Francisco, CA 94158, USA; Memory and Aging Center, Department of Neurology, University of California in San Francisco, San Francisco, CA 94158, USA; Department of Otolaryngology—Head and Neck Surgery, Massachusetts Eye and Ear and Harvard Medical School, Boston, MA 02114, USA

**Keywords:** semantic variant of primary progressive aphasia, speech production, semantics, functional connectivity, compensation mechanism

## Abstract

Semantic variant primary progressive aphasia is a clinical syndrome characterized by marked semantic deficits, anterior temporal lobe atrophy and reduced connectivity within a distributed set of regions belonging to the functional network associated with semantic processing. However, to fully depict the clinical signature of semantic variant primary progressive aphasia, it is necessary to also characterize preserved neural networks and linguistic abilities, such as those subserving speech production. In this case-control observational study, we employed whole-brain seed-based connectivity on task-free MRI data of 32 semantic variant primary progressive aphasia patients and 46 healthy controls to investigate the functional connectivity of the speech production network and its relationship with the underlying grey matter. We investigated brain-behaviour correlations with speech fluency measures collected through clinical tests (verbal agility) and connected speech (speech rate and articulation rate). As a control network, we also investigated functional connectivity within the affected semantic network. Patients presented with increased connectivity in the speech production network between left inferior frontal and supramarginal regions, independent of underlying grey matter volume. In semantic variant primary progressive aphasia patients, preserved (verbal agility) and increased (articulation rate) speech fluency measures correlated with increased connectivity between inferior frontal and supramarginal regions. As expected, patients demonstrated decreased functional connectivity in the semantic network (dependent on the underlying grey matter atrophy) associated with average nouns' age of acquisition during connected speech. Collectively, these results provide a compelling model for studying compensation mechanisms in response to disease that might inform the design of future rehabilitation strategies in semantic variant primary progressive aphasia.

## Teaser

Montembeault *et al.* report that patients with the semantic variant of primary progressive aphasia present with increased functional connectivity within the speech production network, which correlates with preserved verbal agility and increased articulation rate. This study highlights a potential functional mechanism behind preserved speech fluency in these patients.

## Graphical Abstract

**Graphical abstract:**
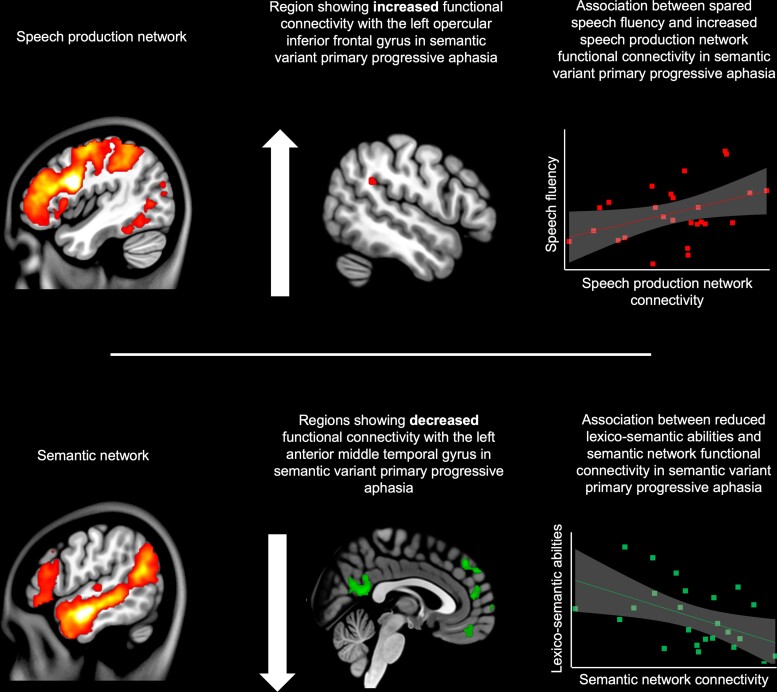

## Introduction

Patients with the semantic variant of primary progressive aphasia (svPPA) present with impaired confrontation naming, single-word comprehension and object knowledge, as well as spared speech production and repetition.^1,2^ Neuroimaging studies have shown an early structural involvement of the left anterior temporal lobe as the epicentre of the disease,^3^ and corresponding decreased functional connectivity across the brain regions anchored to the epicentre (the semantic network).^3–7^ Moreover, the functional decrease of connectivity has been associated with semantic impairments.^4,6^ However, the unique clinical and neuroimaging profile in svPPA is characterized not only by deficits and impaired networks, but also by spared functions and networks. Speech production is a preserved function in svPPA,^8–11^ and neuroimaging studies showed increased functional connectivity across nodes of the speech production network in these patients.^4,6^ However, it has yet to be demonstrated if this increased connectivity in the speech production network is associated with the well-known preserved speech production abilities of these patients, and if it therefore represents compensatory activity in svPPA patients.

The interpretation of an association between increased functional connectivity and spared/enhanced cognitive functions can be challenging. According to Cabeza and colleagues^12^, to be consistent with a compensatory mechanism, brain-behaviour associations require two conditions to be met. First, the enhanced connectivity must be directly or indirectly related to some gap between available neural resources and task demands. Second, the increased connectivity must be related to a beneficial effect on cognitive performance. Considering the disease-related reduction in neural resources that characterizes svPPA patients, we would argue that demonstrating a positive association between preserved speech production and functional connectivity in the speech production network would meet this definition of a compensatory mechanism. Perhaps the greatest hurdle in demonstrating compensatory mechanisms, especially when assessing preserved speech and language abilities, are that patients' performance on such tests tend to be at ceiling or to show limited variability, which in turn affects statistical power. However, with regards to speech production, novel automated connected speech measures have the potential to surmount this issue.^13–15^ This approach entails collecting a speech sample of a few minutes from the patient attempting to speak in complete sentences, for example, while describing a complex picture scene. During such tasks, Cordella and colleagues^9^ have shown that fine-grained measures such as speech rate (number of syllables per millisecond) present great variability in svPPA patients. Thus, such connected speech measures might be optimal to detect brain-behaviour relationships of preserved speech fluency in svPPA patients.

In this study, using a large sample of 32 svPPA patients, we aimed at investigating the associations between functional connectivity of the speech production network and its clinical correlates (verbal agility, speech rate and articulation rate). We will also investigate functional connectivity within the semantic network as a control network. The results of this study will characterize the upregulation and down-regulation in key functional language networks and its association with impaired and preserved speech and language domains in svPPA.

## Methods

### Participants

Forty-four patients with svPPA were recruited at the University of California in San Francisco Memory and Aging Center between 2016 and 2020. They all fulfilled the current diagnostic criteria for imaging-supported svPPA.^1^ Diagnosis was made after a comprehensive evaluation (neurological history and examination, standardized neuropsychological and language assessments) by a multidisciplinary team at a consensus diagnostic meeting at the University of California in San Francisco Memory and Aging Center.

Two groups of healthy controls (HC), who were neurologically and clinically normal as attested by a neurological exam, neuropsychological evaluation and MRI were also included. The first group (*n* = 46) was used as a comparison group for neuroimaging analyses (hereafter called imaging HC). The second group (*n* = 31) was used as a comparison group for behavioural analyses (hereafter called behavioural HC). We used two separate groups of HC to insure sufficient statistical power in each analysis, due to the low number of HC who had both neuroimaging and behavioural data.

All participants gave written consent, and the study was approved by the institutional review board.

### Procedure

#### Cognitive and language assessment procedure

All participants underwent a broad neuropsychological battery and speech and language tests, as previously described.^16,17^ To assess disease severity, we used the clinical dementia rating scale (CDR).^18^

#### Speech fluency and lexico-semantic measures

A picture description task of the Picnic scene from the Western Aphasia Battery^19^ was administered to each participant. They were instructed to describe the picture in as much detail as possible after the following instruction: ‘I'm going to show you a picture. Tell me what you see. Talk in sentences.’ A brief and non-informative prompting was given when they stopped talking for more than a few seconds. Connected speech recordings were performed in clinical settings on a digital video camcorder. The audio files were then manually reviewed and edited to exclude interviewer speech and periods during which the patient was not describing or trying to describe the picture (i.e. asking questions about the task and off-topic comments). ‘Audacity’ was used to reduce background noise from the files through its ‘Noise Reduction’ function. Speech-to-text transcriptions were done manually by trained research assistants. They included everything the participants said (hesitations, half-spoken words, repetitions, self-corrections, incorrect words, pauses fillers, etc.).

To measure speech fluency, we automatically extracted speech rate and articulation rate from the connected speech recording using a script available in Praat based on acoustic features.^20^ Speech rate was calculated as the total number of syllables divided by the total duration of the speech sample in milliseconds (ms). Articulation rate was calculated as the total number of syllables divided by the total phonation time (the duration of speech without pauses) in ms. Therefore, in comparison to speech rate, articulation rate only considers the time when the participant is actively speaking. This measure reduces the impact of word-finding pauses due to the semantic deficit of svPPA patients, which can slow down the overall speech rate. In addition to the connected speech assessment, we also used a brief verbal agility task to characterize speech fluency. In this task, participants must repeat long words (for example, ‘caterpillar’) out loud correctly and as many times as possible within five seconds (total scores ranging from 0 to 6).

To measure lexico-semantics abilities, we automatically extracted the average lexical frequency^21^ and average nouns' age of acquisition (AoA)^22^ from all the nouns included in the connected speech transcriptions using a natural language processing script.^15^ For lexical frequency, we used the SUBTL_WF_ values, which represents the word frequency per million words as calculated from a large corpus of American television and film subtitles.^21^ For AoA, we used subjective word ratings established in a previous psycholinguistic study in which participants were asked to enter the age at which they thought they had learned the word.^22^ In addition to the connected speech assessment, we also used a 16-item version of the Peabody Picture Vocabulary Test (PPVT). In this task, participants must pick one of the four pictures that matches with the word they hear.

#### Neuroimaging

##### MRI protocol

Participants were scanned with a Siemens 3-Tesla Prisma scanner using a body transmit coil and a 64-channel receive head coil. The neuroimaging protocol included a high-resolution MRI structural scan for inter-subject registration, as well as an echoplanar imaging (EPI) scan to study task-free functional connectivity. The structural MRI included a T_1_-weighted 3D magnetization prepared rapid acquisition gradient echo acquired with 160 sagittal slices, TE/TR/TI = 2.9/2300/900 ms, flip angle = 9°, isotropic voxel with size of 1 mm, field-of-view = 256 × 256 mm^2^, matrix = 256 × 256. For task-free functional MRI (fMRI), we acquired 560 T_2_-weighted EPI volumes in interleaved order consisting in 66 anterior commissure/posterior commissure-aligned axial slices with the following parameters: TR/TE = 850/32.8 ms, flip angle = 45°, voxel size = 2.2 × 2.2 × 2.2 mm^3^, field-of-view = 211 × 211 mm^2^, matrix = 96 × 96 in plane resolution and multi-band accelerating factor = 6. Total acquisition time of the task-free fMRI was 8 and 5 s; participants were instructed to remain still and keep their eyes closed without falling asleep. Two spin-echo volumes, one with anterior-to-posterior and one with posterior-to-anterior phase-encoding, were also acquired for susceptibility-induced distortion correction.

##### Estimation of grey matter volume

Structural MRI data were preprocessed using the Computational Anatomy Toolbox (CAT12; dbm.neuro.uni-jena.de/cat) running under MATLAB version R2017b. The CAT12 classifies T_1_-weighted data as grey matter (GM), white matter or cerebrospinal fluid using an improved segmentation approach compared to the traditional unified segmentation,^23^ based on an adaptive maximum a posteriori. GM probability maps were then nonlinearly normalized to the Montreal Neurologic Institute (MNI) space using Diffeomorphic Anatomical Registration Through Exponentiated Lie Algebra,^24^ and modulated by the Jacobian determinant of the deformations derived from the spatial normalization. We used these GM maps to extract disease-severity related covariates of no interest for the task-free fMRI analyses. Furthermore, to better characterize the patient group in relation to previous studies, we examined global atrophy patterns in svPPA patients by comparing GM probability maps in svPPA versus HC. To this end, modulated GM images were smoothed with an isotropic Gaussian kernel of 6 mm full width at half maximum (FWHM). Voxel-based inferential statistic is described in section ‘Neuroimaging analyses’.

##### Preprocessing of task-free fMRI

The preprocessing pipeline for the functional connectivity analysis was implemented in Python and made use of tools available in Functional Magnetic Resonance Imaging of the Brain Software Library (FSL), Statistical Parametric Mapping (SPM), Advanced Normalization Tools (Avants *et al*., 2010, 2011) and Analysis of Functional NeuroImaging (Cox, 1996; Cox & Hyde, 1997; Gold *et al*., 1998; https://afni.nimh.nih.gov/). The first five volumes of the acquisition were discarded to allow T_1_ equilibrium to be established. The remaining 555 volumes were slice-time corrected, realigned to the mean functional image and assessed for rotational and translational head motion. Susceptibility-induced distortions characteristic of EPI acquisitions were estimated and corrected using the TOPUP tool in FSL using the two spin-echo images acquired with opposing polarities of the phase-encode blips. The functional volumes were then linearly registered to an EPI template created from the mean functional images of the participants enrolled in this study. The EPI template was then normalized to the ICBM2009 MNI space using a combination of linear and non-linear warping, and the transformations were subsequently applied to the task-free fMRI data, producing MNI-normalized functional volumes with a 2 × 2 × 2 mm^3^ resolution. These volumes were spatially smoothed with a 5-mm FWHM Gaussian kernel. Cerebrospinal fluid and white matter tissue probability maps were then used to compute the mean time-series used as regressors. Functional data were then bandpass-filtered (0.008 Hz < f < 0.15 Hz), and the nuisance variables were regressed out from the data, which included the six motion parameters, the first derivative and quadratic terms, as well as cerebrospinal fluid and white matter time series as suggested in the study of Satterthwaite *et al*.^25^

Subjects were included only if they met all the following criteria: no inter-frame head translations >2 mm, no inter-frame head rotations >2° and <28 motion spikes (defined as inter-frame head displacements >1 mm), which represents 5% of the total number of frames. Based on these criteria, three svPPA patients were excluded because of excessive motion and nine svPPA patients were excluded because of poor data acquisition and normalization to the standard template. None of the healthy controls were excluded. Thus, the final cohort consisted of 32 svPPA patients and 46 healthy controls (HC1). To assess differences in head motion between svPPA patients and healthy participants, we calculated the root mean square (RMS) of the six motion parameters—including three translations and three rotations—along the *xyz* axes in each group. RMS mean ± standard values were as follows: healthy participants (0.11 ± 0.05), svPPA (0.13 ± 0.06). A two-sample *t*-test found no significant differences in RMS motion values across the two groups (*T* = 1.6, *P* = 0.12).

##### Definition of the seed regions of interest

**Figure 1:**
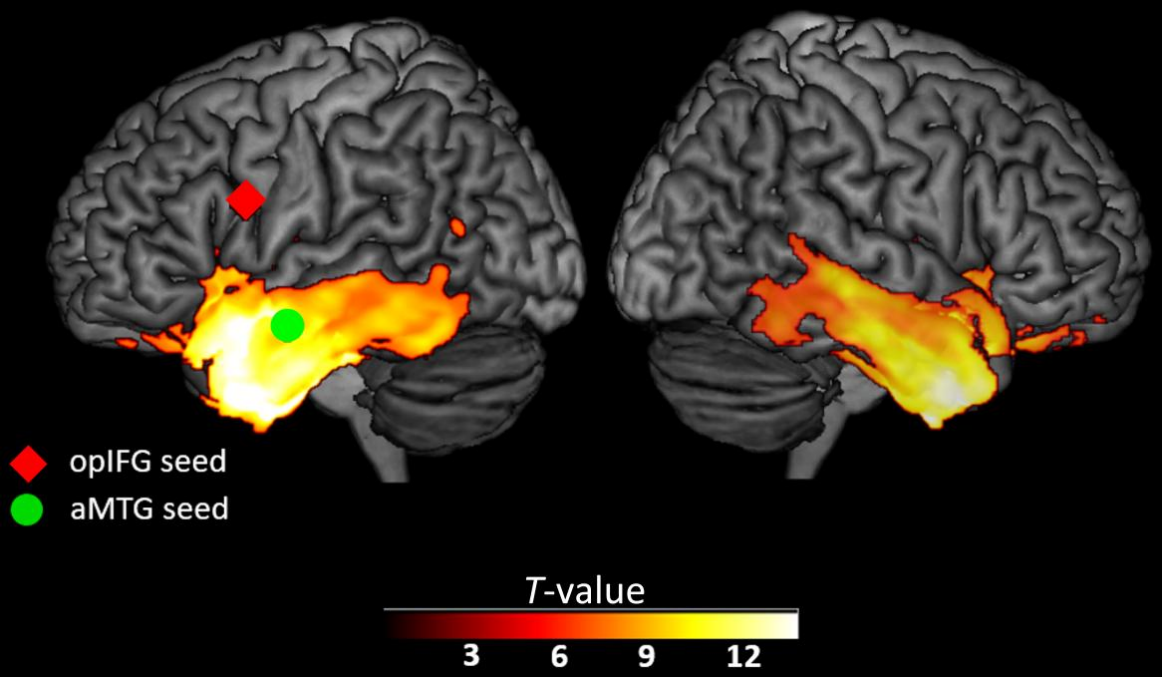
**Pattern of atrophy in svPPA patients and seed regions for functional connectivity analyses.** The statistical map was thresholded at *P* (FWE) < 0.05 and is shown on a rendered surface of the Montreal Neurological Institute (MNI) template. Colorbar represents *T*-score. aMTG = anterior middle temporal gyrus; opIFG = opercular inferior frontal gyrus.

We defined two seed regions to extract the speech production network and the control network, i.e. the semantic network (Fig. 1).

The seed ROI for the speech production network was defined as a sphere of 4-mm radius centred in the pars opercularis of the inferior frontal gyrus (opIFG; MNI coordinates: *x* = −50, *y* = 8, *z* = 23) as described in the study of Battistella *et al.*^4,26^ The MNI coordinates of the seed in the left opIFG were identified in a previous study by contrasting a phonemic fluency task against semantic and syntactic fluency tasks.^27^

The seed ROI for the semantic network was defined as a sphere of 4-mm radius centred in the left anterior middle temporal gyrus (aMTG; MNI coordinates: *x* = −60, *y* = −6, *z* = −18).^4,26^ It was identified in a previous study by contrasting a semantic association task on pairs of famous faces against a perceptual matching task related to pairs of unknown faces.^28^

##### Seed-based functional connectivity analysis

Single-subject correlation maps were generated by calculating the *r*–Pearson correlation coefficient between the average blood-oxygen-level-dependent signal time course from the seed ROIs and the time course from all other voxels of the brain. Correlation maps were converted to *z*-scores to enable parametric statistical comparisons.

### Statistical analyses

#### Behavioural scores analysis

We conducted independent two-sample *t*-tests controlling for age, sex and education, to compare speech fluency and lexico-semantics features between HC and svPPA patients. Given the significant correlations amongst speech fluency measures and lexico-semantic measures, we set the threshold for these analyses at *P* < 0.05 uncorrected.

#### Neuroimaging analyses

##### Voxel-based morphometry: GM comparisons

Voxel-based inferential statistic was performed by fitting a general linear model in SPM12 on the smoothed and modulated GM tissue probability maps entering age, sex and total GM volume as covariates of no interest. The statistical map showing GM volume differences between HC and svPPA was thresholded at *P* < 0.05 family wise error (FWE) corrected.

##### Task-free fMRI: functional network comparisons

We identified the two functional language networks of interest (hereafter called intrinsic functional connectivity networks, ICN) using a one-sample *t*-test with sex and age as covariates of no interest on the correlation maps extracted from patients and HC and converted to *z*-scores. Statistical thresholds on the resulting group-level connectivity maps were applied at *P* < 5 × 10^−5^ after peak-level FWE correction for multiple comparisons over the whole brain. To determine functional connectivity changes in svPPA patients compared to HC in each of the two networks, we used voxel-wise two-sample *t*-test in SPM12 on the single-subject connectivity maps using age, sex, education and RMS values as covariates of no interest. We included an implicit mask of the ICNs defined at group level (connectivity in both patients and HC) to restrict the significant clusters to the networks under examination. Results were thresholded at *P* < 0.05, cluster-level FWE corrected. To assess whether observed group differences could be related to underlying GM atrophy, we re-estimated the models to include also total GM volume and the GM volume of the corresponding seed ROIs as covariates of no interest (Battistella *et al*., 2019).

##### Brain-behaviour correlation

In svPPA patients, we performed correlation analyses between clusters showing increased or decreased connectivity in svPPA versus HC and relevant speech/language scores (section ‘Speech fluency and lexico-semantic measures’). The relationships between the behavioural variables and the functional connectivity were determined using Pearson partial correlations removing the effect of disease severity in each correlation by controlling for CDR and RMS values derived from the pre-processing of task-free fMRI data. Given the significant correlations amongst speech fluency measures, and amongst lexico-semantic measures (Supplementary Table 1), we set the threshold at *P* < 0.05 uncorrected for these analyses.

### Data availability

While we can share anonymized data, public archiving is not yet permitted under the study's institutional review board approval due to the sensitive nature of patient data. Specific requests can be submitted through the University of California in San Francisco—Memory and Aging Center Resource (Request form: http://memory.ucsf.edu/resources/data). Following a University of California in San Francisco-regulated procedure, access will be granted to designated individuals in line with ethical guidelines on the reuse of sensitive data. This would require submission of a Material Transfer Agreement, available at: https://icd.ucsf.edu/material-transfer-and-data-agreements. Commercial use will not be approved.

## Results

### Description of participants

The summary of the demographic and clinical presentation of svPPA patients and behavioural HC is included in Table 1. There was no difference between behavioural HC and svPPA at the demographic level. SvPPA patients presented the expected profile of impaired naming, single-word comprehension and irregular word reading. They additionally presented with verbal short-term memory, sentence comprehension, visual episodic recall (but not recognition) and executive deficits. Verbal working memory, visuospatial and visuoconstructive abilities were preserved.

**Table 1:** Demographics, language and cognitive data

	svPPA (*n* = 32)	HC (*n* = 31)
**Demographics**		
Sex (M/F)	14/18	11/20
Age	67.9 **±** 6.5	69.3 ± 8.5
Years of education	15.8 **±** 2.5	16.9 ± 1.3
Handedness (RH/non-RH)	30/2	22/9
Disease characteristics		
Mini-Mental State Examination (/30)	22.1 **±** 6.2***	29.7 ± 0.8
Clinical Dementia Rating Total	1.0 **±** 0.6***	0.0 ± 0.0
Clinical Dementia Rating Box score	5.0 **±** 3.0***	0.0 ± 0.0
Language		
Boston Naming Test (/15)	5.4 ± 4.2***	14.8 ± 0.4
Irregular words reading (/6)	4.7 ± 1.5***	5.9 ± 0.3
Sentence comprehension (5)	4.4 ± 1.0*	4.9 ± 0.3
Repetition (/5)	4.0 ± 1.2**	4.9 ± 0.3
Apraxia of speech (/7)	0.0 ± 0.2	0.0 ± 0.0
Cognition		
VOSP Number location (/10)	8.5 ± 2.5	8.7 ± 1.4
Benson Figure Copy (/17)	15.3 ± 1.4	15.7 ± 1.4
Benson Figure Recall (/17)	5.6 ± 4.4***	12.8 ± 3.2
Benson Figure Recognition (/1)	0.8 ± 0.4	0.9 ± 0.3
Digit span forwards	6.0 ± 1.5*	7.2 ± 1.1
Digit span backwards	5.0 ± 1.5	5.0 ± 1.1
Modified Trails (seconds)	55.9 ± 34.0***	26.1 ± 11.4
Stroop Inhibition (correct)	40.4 ± 17.8**	55.3 ± 10.5
Design fluency (correct)	7.3 ± 3.6***	12.1 ± 3.1
Letter fluency	7.5 ± 4.7***	17.7 ± 4.4
Category fluency	9.6 ± 7.4***	24.2 ± 4.3
Speech fluency measures		
Verbal agility task (/6)	5.1 ± 1.1	5.6 ± 0.7
Speech rate	2.6 ± 1.0	2.6 ± 0.6
Articulation rate	4.6 ± 1.0***	3.8 ± 0.5
Lexico-semantic measures		
Peabody Picture Vocabulary Test (PPVT;/16)	9.8 ± 4.2***	15.7 ± 0.7
Average nouns frequency	4.8 ± 0.9***	3.9 ± 0.3
Average nouns' AoA	4.3 ± 0.6***	4.9 ± 0.3

Significant differences between behavioural HC and svPPA are denoted as: * *P* < 0.05; ** *P* < 0.01; *** *P* < 0.001; F = female; M = male; non-R = non right-handed; PPVT = Peabody Picture Vocabulary Test; RH = right-handed; VOSP = Visual Object and Space Perception battery.

The imaging HC group included 46 participants (male/female: 16/30; mean age = 67.5 **±** 7.0; mean years of education: 17.4 **±** 1.8; right-handed/non-right-handed: 40/6). All demographic variables were comparable between the two groups, except for years of education. We therefore controlled for education in our analyses (in addition to the other variables described in Statistical analyses). The VBM analysis revealed the expected pattern of atrophy in svPPA, involving the bilateral medial and lateral temporal lobes, as well as the bilateral insula (Fig. 1).

### Behavioural and imaging findings related to speech production

#### Behavioural measures

**Figure 2:**
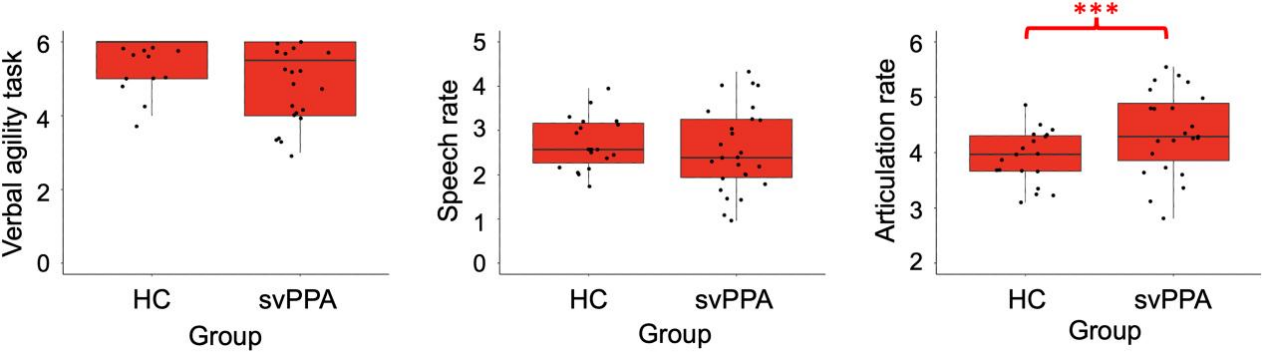
**Comparison of speech fluency measures between svPPA patients and HC.** Independent two-sample *t*-tests controlling for age, sex and education were conducted (* *P* < 0.05; ** *P* < 0.01; *** *P* < 0.001). The verbal agility task is scored out of 6 points. Speech rate and articulation rate are presented as syllables per milliseconds. Individual points represent the values for each subject.

SvPPA patients showed preserved or better performance on measures of speech fluency. Articulation rate was significantly greater in svPPA versus HC (*P* < 0.001; Fig. 2). Speech rate and verbal agility were equivalent between svPPA and HC (*P* = 0.577 and *P* = 0.280, respectively, Fig. 2).

#### Functional connectivity

**Figure 3:**
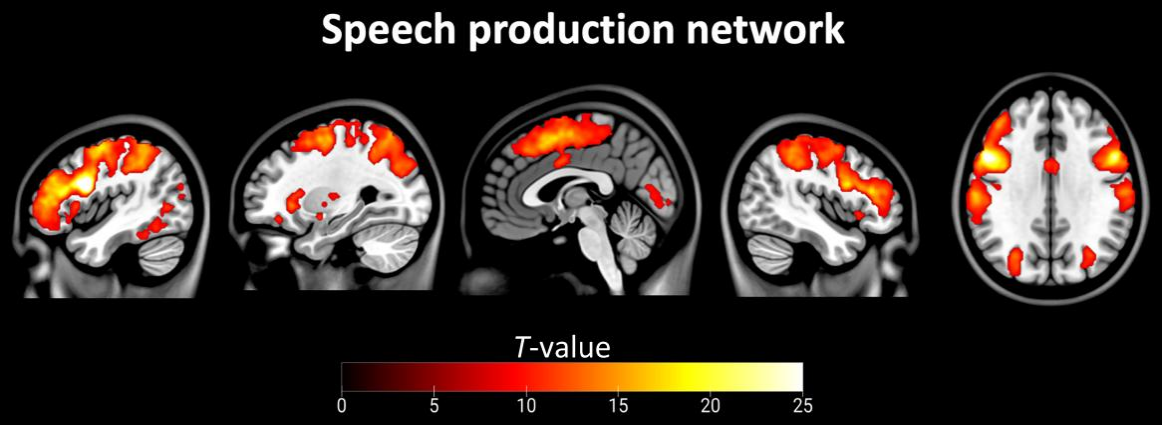
**Speech production network connectivity in HC and svPPA patients.** The network was identified using a one-sample *t*-test with sex and age as covariates of no interest on the correlation maps extracted from patients and HC and converted to *Z*-scores. Statistical thresholds on the resulting group-level connectivity maps were applied at *P* < 5 × 10^−5^ after peak-level FWE correction for multiple comparisons over the whole brain. Statistical maps are superimposed on a series of slices of the standard brain in the MNI space. Colorbar represents *T*-score.

**Figure 4:**
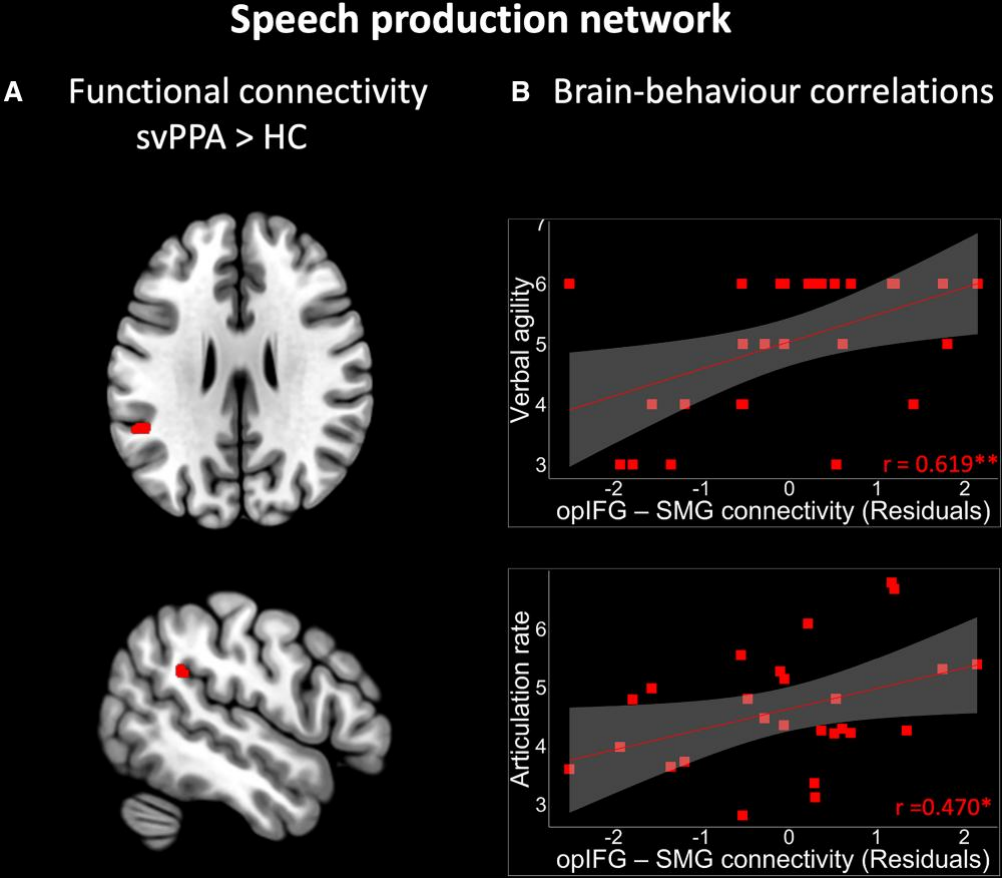
**Differences in functional connectivity in the speech production network and associated correlation with speech fluency scores. (A)** Comparison of functional connectivity between svPPA patients and HC in the speech production network. A voxel-wise two-sample *t*-test in SPM12 on the single-subject connectivity maps was used using age, sex, education and RMS as covariates of no interest. We included an implicit mask of the ICNs defined at group level (connectivity in both patients and HC) to restrict the significant clusters to the networks under examination. Results were thresholded at *P* < 0.05, cluster-level FWE corrected. To assess whether observed group differences could be related to underlying GM atrophy, we re-estimated the models to include also total GM volume and the GM volume of the corresponding seed ROIs as covariates of no interest. The cluster showing increased connectivity in svPPA versus HC is shown in red. (**B**) Significant brain-behaviour correlations between opIFG-SMG functional connectivity strength (residual values after removing the effect of disease severity as measured with the CDR and subjects' motion during the task-free fMRI as measured with the RMS) and speech fluency measures in svPPA, as measured using Pearson partial correlations. Line represents the fitting of the distribution of the values; *R* correlation coefficient is shown at the bottom right of each plot (*P* < 0.05*; *P* < 0.01**; *P* < 0.001***). opIFG = Inferior frontal gyrus pars opercularis; SMG = Supramarginal gyrus.

The speech production network anchored to the left opIFG seed included areas in the bilateral opercular and triangular part of the inferior frontal gyrus, left middle frontal gyrus, bilateral supramarginal gyrus and inferior parietal sulcus, left putamen, left pre supplementary motor area and left inferior temporal gyrus, as previously described^26^ (Fig. 3, Supplementary Table 2). Within this ICN, svPPA patients showed increased functional connectivity in comparison to HC (Fig. 4A). The significant difference was observed in the left supramarginal gyrus (SMG; MNI coordinates of the cluster peak: −52, −46, 28; *T*-value: 5.1; cluster size: 33 voxels). The increased connectivity in the opIFG-seeded ICN remained significant when controlling for measures of GM volume. Within this ICN, we did not find any regions showing decreased functional connectivity in svPPA compared to HC.

#### Brain-behaviour correlations

In svPPA patients, functional connectivity between the left opIFG and left SMG was significantly correlated with two of the three speech fluency measures, namely verbal agility (*r* = 0.619 *P* = 0.005) and articulation rate (*r* = 0.470, *P* = 0.042) (Fig. 4B), but not with speech rate (*r* = 0.285 *P* = 0.237). Conversely, functional connectivity within the speech production network did not significantly correlate with lexico-semantic measures (PPVT: *r* = 0.130 *P* = 0.597; average nouns lexical frequency *r* = −0.013 *P* = 0.957; average nouns' AoA: *r* = 0.049 *P* = 0.843).

### Control network: behavioural and imaging findings related to semantics

#### Behavioural measures

SvPPA patients showed impaired performance on all lexico-semantic measures. Average nouns frequency, average nouns' AoA and single-word comprehension were lower in svPPA versus HC (*P* < 0.001).

#### Functional connectivity

**Figure 5:**
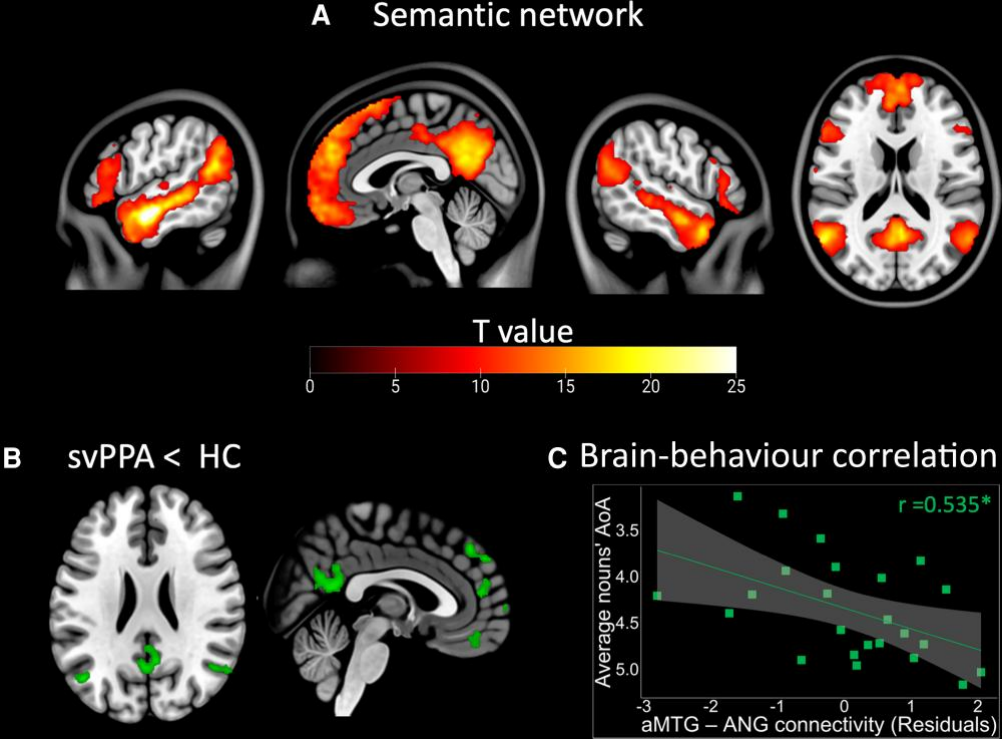
**Semantic network connectivity and associated correlation with linguistic scores.** (**A**) Semantic network maps in HC and svPPA patients. The network was identified using a one-sample *t*-test with sex and age as covariates of no interest on the correlation maps extracted from patients and HC and converted to *Z*-scores. Statistical thresholds on the resulting group-level connectivity maps were applied at *P* < 5 × 10^−5^ after peak-level FWE correction for multiple comparisons over the whole brain. Statistical maps are superimposed on a series of sagittal slices of the standard brain in the MNI space. Colorbar represents *T*-score. (**B**) Comparison of functional connectivity between svPPA patients and HC in the semantic network. A voxel-wise two-sample *t*-test in SPM12 on the single-subject connectivity maps was used using age, sex, education and RMS as covariates of no interest. We included an implicit mask of the ICNs defined at group level (connectivity in both patients and HC) to restrict the significant clusters to the networks under examination. Results were thresholded at *P* < 0.05, cluster-level FWE corrected. To assess whether observed group differences could be related to underlying GM atrophy, we re-estimated the models to include also total GM volume and the GM volume of the corresponding seed ROIs as covariates of no interest. The clusters showing decreased connectivity in svPPA versus HC are shown in green. (**C**) Significant brain-behaviour correlation between aMTG-AG functional connectivity strength (residual values after removing the effect of disease severity as measured with the CDR and subjects' motion during the task-free fMRI as measured with the RMS) and one of the lexico-semantic measure (average nouns' AoA) in svPPA, as measured using a Pearson partial correlation. Line represents the fitting of the distribution of the values; *R* correlation coefficient is shown at the bottom right of the plot (*P* < 0.05*; *P* < 0.01**; *P* < 0.001***). aMTG = anterior middle temporal gyrus; ANG = angular gyrus; AoA = age of acquisition.

The semantic ICN anchored to the left aMTG seed included areas in the bilateral middle temporal gyrus, temporal pole, inferior temporal gyrus, precuneus, hippocampus and parahippocampal gyrus in HC. Additional significant regions were located in the left angular gyrus, anterior insula, orbital inferior frontal gyrus, anterior and posterior cingulate cortices, as well as in the right middle occipital, and orbital medial frontal gyrus^26^ (Fig. 5A, Supplementary Table 2). SvPPA patients showed decreased functional connectivity in the aMTG-seeded network in comparison to HC (Fig. 5B). The significant differences were observed bilaterally in the angular gyrus (AG) (MNI coordinates of the clusters' peak: −46, −70,26 and 54, −62,26; *T*-value: 5.0 and 4.2; cluster size: 70 and 142 voxels), as well as anterior and posterior cingulate cortices (MNI coordinates of clusters' peaks: 6,48,14 and 4, −50,18; *T*-value: 5.0 and 4.8; cluster size: 136 and 388 voxels). The decreased connectivity in the aMTG-seeded ICN was no longer significant when controlling for measures of GM volume. Within this ICN, we did not find any regions showing increased functional connectivity in svPPA compared to HC.

#### Brain-behaviour correlations

Functional connectivity between the left aMTG and left angular gyrus was significantly correlated with average nouns' AoA in svPPA patients (*r* = 0.535 *P* = 0.018; Fig. 5C). However, it did not significantly correlate with PPVT (*r* = 0.146; *P* = 0.551) or average nouns lexical frequency (*r* = −0.399; *P* = 0.091). In addition, functional connectivity within the semantic network did not significantly correlate with speech fluency measures (verbal agility: *r* = 0.192 *P* = 0.432; speech rate: *r* = 0.070 *P* = 0.777; articulation rate: *r* = 0.099 *P* = 0.685).

### Post-hoc analyses: the compensatory role of the increased connectivity in the speech production network

We performed post-hoc analyses to further investigate the specificity of the correlation between speech fluency scores and connectivity within the speech production network. Specifically, we tested if the increased speech production network functional connectivity observed in svPPA could be associated with (i) disease severity and (ii) other preserved cognitive abilities in svPPA. To answer the first question, we assessed the correlation between functional connectivity in the speech production network with two measures of disease severity: Mini-Mental State Examination and CDR. No significant correlations were observed (*r* = 0.200 *P* = 0.281; *r* = −0.047 *P* = 0.802, respectively). To answer the second question, we assessed the correlation with all cognitive tests that showed preserved performance in svPPA (Table 1): the number location subtest from the Visual Object and Space Perception battery, Benson Figure copy, Benson Figure recognition and Digit span backwards. No significant correlations were observed with any of those tests (Number location: *r* = 0.296 *P* = 0.134; Benson Figure copy: *r* = 0.069 *P* = 0.717; Benson Figure recognition: *r* = 0.164 *P* = 0.404; Digit span backwards: *r* = −0.024 *P* = 0.904).

## Discussion

In this study, we found that increased functional connectivity in the speech production network correlated with articulation rate, a measure of speech fluency in svPPA. This observed relationship was highly specific to speech fluency measures, as it was not observed with lexico-semantic, general cognition or other preserved cognitive scores. In contrast, we observed that svPPA patients presented with decreased functional connectivity in the semantic network, highlighting the interplay between functional connectivity networks subserving spared and impaired language domains in svPPA.

Our study reveals speech production-related compensation mechanisms occurring in svPPA. Facing severe atrophy in ATL regions associated with linguistic and semantic deficits, svPPA patients show increased connectivity in the speech production network that is correlated with a beneficial effect on cognitive performance (i.e. spared verbal agility and increased articulation rate). Our results therefore fulfil the two required criteria to establish the presence of a compensation mechanism.^12^ It is important to note that in this case, the beneficial effect of this compensatory activity is not observed on the core semantic impairment. Nonetheless, our results suggest that the upregulation of the speech production network might allow svPPA patients to speak at a normal or even faster speed. Conversely, patients with the non-fluent/agrammatic variant of primary progressive aphasia, who show down-regulation of the speech production network, present with predominant speech fluency deficits.^29^ The preserved speech rate and increased articulation rate in svPPA are particularly interesting, given that decreases in speech rates have been reported in logopenic variant of primary progressive aphasia, as well as in non-speech/language predominant neurodegenerative diseases such as Alzheimer's disease and behavioural variant of frontotemporal dementia.^30,31^ Few other studies have reported increased speech fluency in svPPA patients, which were interpreted as either anxiety and/or disinhibition^32^ or as an increased tendency for logorrhoea and circumlocution.^33^ In the present study, speech and articulation rates during connected speech were not associated with the use of nouns that are more frequent or acquired earlier in life. Nonetheless, future studies should clarify the underlying mechanism of increased articulation rate in svPPA patients, for example by investigating its associations with neuropsychiatric symptoms or other linguistic changes such as the use of fillers or functional words, which are usually elevated in svPPA.^34–36^ The observed compensation mechanism related to speech production could be related to positive benefits in the daily life of svPPA patients. For example, svPPA patients remain active participants in conversations, taking an equal proportion of turns as their relatives^37^ and sharing more autobiographical stories than HC.^38^ Comparatively, a reduced conversational speech output has been reported in most other neurodegenerative diseases.^38–41^ However, it is important to acknowledge that this preserved or increased participation in social interactions in svPPA can also come with less positive outcomes, such as more conversations breakdowns, perseverations on aspects of self and failure to attend to social cues.^38,39^

To our knowledge, this is the first report of compensation mechanisms in svPPA, occurring in the form of functional connectivity within an ICN. Previously, task-based imaging studies have reported potential compensation mechanisms in svPPA. In a svPPA case study using magnetoencephalography, preserved behavioural performance on a general-level semantic categorization task was associated with hyperactivation of the left inferior temporal gyrus and right ATL.^42^ Similarly, in a larger magnetoencephalography study, it was reported that svPPA patients showed an over-recruitment of perceptual processing brain regions, while presenting preserved performance on a shallow semantic categorization task.^43^ Two case series also showed that in some svPPA patients, cortical hyperactivations might be associated with preserved episodic future thinking and autobiographical memory;^44,45^ however, this effect was not observed in all patients, depending on their atrophy patterns. Other studies have also reported increased functional activations in svPPA, but they had not demonstrated any positive impacts on cognition.^46–49^ Altogether, our study and these past studies highlight the functional reorganization that occurs in the face of neurodegenerative processes associated with svPPA. Our study adds to the existing literature within neurological disorders in which decreased functional connectivity in one specific network is accompanied by increased functional connectivity in another, highlighting the importance of investigating not only affected but also spared networks .^50–54^

Our study also highlights the potential of automated approaches to investigate impaired and spared speech and language domains in svPPA. Automated approaches such as natural language processing and acoustic analyses allow for the quick extraction of features characterizing multiple speech and language domains and in a manner that might be more objective.^9^ Automated approaches are particularly useful in centres where there is limited or no access to speech and language pathologists. Specifically, our study highlights the particular importance of employing fine-grained measures of speech fluency, which can differentiate the influence of pausing versus articulatory mechanisms on speech rate. For example, in the current study, speech rate (which can be influenced by both pauses and articulator movement) was similar between svPPA and HC, while articulation rate (which removes the impact of pausing) was increased in svPPA compared to HC. These observations are clinically meaningful, since spared speech production is part of the diagnostic criteria for svPPA and informs on the differential diagnosis with other variants. Future studies should investigate speech production in more severe svPPA cases as well as in a longitudinal follow-up.

The results from this study could also inform on the development and selection of interventions for patients with svPPA. Indeed, spared or enhanced cognitive or linguistic functions are often used when intervening with neurodegenerative patients to support impaired functions.^55,56^ Leveraging spared speech production abilities is no exception. For example, previous studies have proven that lexical retrieval treatment, in which the svPPA patients develop self-cueing strategies to promote naming, demonstrate strong direct treatment effects, 1-year maintenance of gains and generalization to untrained items.^57^ This intervention capitalizes on spared speech production to facilitate word retrieval by training the individuals to verbally circumlocute by providing semantic features (ex: ‘It is black’ or ‘It is made of plastic’) or episodic/autobiographic information (ex: ‘I use this when I prepare my tea every afternoon’) when having word-finding difficulty.

Finally, the decrease of functional connectivity in the semantic network in our study supports the hypothesis of network-wide selective vulnerability to focal neurodegeneration.^3,58,59^ Investigating functional connectivity in networks where one or several nodes are structurally damaged is particularly challenging, as even by statistically removing the effect of atrophy in connectivity measures, it remains possible that structural neurodegeneration still affects network-wide intrinsic connectivity. Thus, it is hard to disentangle whether regions of altered connectivity within a specific brain network precede neuropathology or are a consequence of focal degeneration. Here, we showed that the decreased connectivity in the semantic network between the left aMTG and the angular gyrus in svPPA compared to HC is dependent on the degeneration of the temporal region, as those differences were not present when removing the effect of the GM volume in the aMTG. The possibility to investigate spared brain networks and regions distant from the core atrophic region represents an exciting solution to the recurring conundrum of differentiating the effects of neurodegeneration from brain abnormalities preceding brain atrophy. Thus, changes in spared networks (i.e. connectomal diaschisis^60^) may constitute a powerful tool to inform the design of rehabilitation strategies, such as neuromodulation approaches, in svPPA. Overall, the pattern of brain connectivity revealed in this study is consistent with previous studies^4,6^ and highlights that the damage of the ventral language network and upregulation of the dorsal language network in svPPA is a central feature of this condition.^61^ Nonetheless, the present analyses do not provide information on the directionality of the functional connectivity between the nodes of the speech production and semantic networks, and as such future studies should use dynamic causal modelling to better understand the interplay between these two networks.

In conclusion, the current study highlights that even in the presence of severe neurodegeneration, large-scale networks can show increased functional connectivity that relates to specific spared cognitive functions. These findings provide a compelling framework for studying compensation mechanisms in response to disease that might inform the design of rehabilitation strategies in this population.

## Supplementary Material

fcad077_Supplementary_Data

## Abbreviations

aMTG =: anterior middle temporal gyrus
ANG =: angular gyrus
ANTs =: Advanced Normalization Tools
AFNI =: Analysis of Functional NeuroImaging
AoA =: age of acquisition
BOLD =: blood-oxygen-level-dependent
CAT12 =: Computational Anatomy Toolbox 12
CDR =: clinical dementia rating scale
DARTEL =: Diffeomorphic Anatomical Registration Through Exponentiated Lie Algebra
EPI =: echoplanar imaging
fMRI =: functional magnetic resonance imaging
FSL =: Functional Magnetic Resonance Imaging of the Brain Software Library
FWE =: family-wise error
FWHM =: full width at half maximum
GM =: grey matter
HC =: healthy controls
ICN =: intrinsic functional connectivity network
MNI =: Montreal Neurologic Institute
MPRAGE =: magnetization prepared rapid acquisition gradient echo
opIFG =: Inferior frontal gyrus pars opercularis
PPVT =: Peabody Picture Vocabulary Test
RMS =: root mean square
ROI =: region of interest
SMG =: Supramarginal gyrus
SPM12 =: Statistical Parametric Mapping 12
svPPA =: semantic variant of primary progressive aphasia

## References

[1] Gorno-Tempini ML , Hillis AE, Weintraub S, et al Classification of primary progressive aphasia and its variants. Neurology. 2011;76(11):1006–1014. 21325651 10.1212/WNL.0b013e31821103e6 PMC3059138

[2] Montembeault M , Brambati SM, Gorno Tempini ML, Migliaccio R. Clinical, anatomical and pathological features in the three variants of primary progressive aphasia: A review. Front Neurol. 2018;9:692. 30186225 10.3389/fneur.2018.00692 PMC6110931

[3] Guo CC , Gorno-Tempini ML, Gesierich B, et al Anterior temporal lobe degeneration produces widespread network-driven dysfunction. Brain. 2013;136(Pt 10):2979–2991. 24072486 10.1093/brain/awt222 PMC3857932

[4] Battistella G , Henry M, Gesierich B, et al Differential intrinsic functional connectivity changes in semantic variant primary progressive aphasia. NeuroImage Clin. 2019;22:101797. 31146321 10.1016/j.nicl.2019.101797 PMC6465769

[5] Bonakdarpour B , Hurley RS, Wang AR, et al Perturbations of language network connectivity in primary progressive aphasia. Cortex. 2019;121:468–480. 31530376 10.1016/j.cortex.2019.08.010 PMC6889875

[6] Montembeault M , Chapleau M, Jarret J, et al Differential language network functional connectivity alterations in Alzheimer's disease and the semantic variant of primary progressive aphasia. Cortex. 2019;117:284–298. 31034993 10.1016/j.cortex.2019.03.018

[7] Meijboom R , Steketee RM, Ham LS, van der Lugt A, van Swieten JC, Smits M. Differential hemispheric predilection of microstructural white matter and functional connectivity abnormalities between respectively semantic and behavioral variant frontotemporal dementia. J Alzheimer's Dis. 2017;56(2):789–804. 28059782 10.3233/JAD-160564

[8] Ash S , Nevler N, Phillips J, et al A longitudinal study of speech production in primary progressive aphasia and behavioral variant frontotemporal dementia. Brain Lang. 2019;194:46–57. 31075725 10.1016/j.bandl.2019.04.006 PMC6656376

[9] Cordella C , Dickerson BC, Quimby M, Yunusova Y, Green JR. Slowed articulation rate is a sensitive diagnostic marker for identifying non-fluent primary progressive aphasia. Aphasiology. 2017;31(2):241–260. 28757671 10.1080/02687038.2016.1191054 PMC5531197

[10] Leyton CE , Hodges JR, Piguet O, Ballard KJ. Common and divergent neural correlates of anomia in amnestic and logopenic presentations of Alzheimer's disease. Cortex. 2017;86(Supplement C):45–54. 27875715 10.1016/j.cortex.2016.10.019

[11] Wilson SM , Henry ML, Besbris M, et al Connected speech production in three variants of primary progressive aphasia. Brain. 2010;133(Pt 7):2069–2088. 20542982 10.1093/brain/awq129 PMC2892940

[12] Cabeza R , Albert M, Belleville S, et al Maintenance, reserve and compensation: The cognitive neuroscience of healthy ageing. Nat Rev Neurosci. 2018;19(11):701–710. 30305711 10.1038/s41583-018-0068-2 PMC6472256

[13] Boschi V , Catricalà E, Consonni M, Chesi C, Moro A, Cappa SF. Connected speech in neurodegenerative language disorders: A review. Review. Front Psychol. 2017;8:269. 28321196 10.3389/fpsyg.2017.00269 PMC5337522

[14] Slegers A , Filiou RP, Montembeault M, Brambati SM. Connected speech features from picture description in Alzheimer's disease: A systematic review. J Alzheimer's Dis. 2018;65(2):519–542. 30103314 10.3233/JAD-170881

[15] Slegers A , Chafouleas G, Montembeault M, et al Connected speech markers of amyloid burden in primary progressive aphasia. Cortex. 2021;145:160–168. 34731686 10.1016/j.cortex.2021.09.010 PMC9381031

[16] Gorno-Tempini ML , Dronkers NF, Rankin KP, et al Cognition and anatomy in three variants of primary progressive aphasia. Ann Neurol. 2004;55(3):335–346. 14991811 10.1002/ana.10825 PMC2362399

[17] Kramer JH , Jurik J, Sha SJ, et al Distinctive neuropsychological patterns in frontotemporal dementia, semantic dementia, and Alzheimer disease. Cogn Behav Neurol. 2003;16(4):211–218. 14665820 10.1097/00146965-200312000-00002

[18] Berg L . Clinical dementia rating (CDR). Psychopharmacol Bull. 1988;24(4):637–639. 3249765

[19] Kertesz A . Western aphasia battery test manual. Psychological Corporation; 1982.

[20] de Jong NH , Wempe T. Praat script to detect syllable nuclei and measure speech rate automatically. Behav Res Methods. 2009;41(2):385–390. 19363178 10.3758/BRM.41.2.385

[21] Brysbaert M , New B. Moving beyond kucera and francis: A critical evaluation of current word frequency norms and the introduction of a new and improved word frequency measure for American English. Behav Res Methods. 2009;41(4):977–990. 19897807 10.3758/BRM.41.4.977

[22] Kuperman V , Stadthagen-Gonzalez H, Brysbaert M. Age-of-acquisition ratings for 30,000 English words. Behav Res Methods. 2012;44(4):978–990. 22581493 10.3758/s13428-012-0210-4

[23] Ashburner J , Friston KJ. Unified segmentation. Neuroimage. 2005;26(3):839–851. 15955494 10.1016/j.neuroimage.2005.02.018

[24] Ashburner J . A fast diffeomorphic image registration algorithm. Neuroimage. 2007;38(1):95–113. 17761438 10.1016/j.neuroimage.2007.07.007

[25] Satterthwaite TD , Elliott MA, Gerraty RT, et al An improved framework for confound regression and filtering for control of motion artifact in the preprocessing of resting-state functional connectivity data. Neuroimage. 2013;64:240–256. 22926292 10.1016/j.neuroimage.2012.08.052 PMC3811142

[26] Battistella G , Borghesani V, Henry M, et al Task-Free functional language networks: Reproducibility and clinical application. J Neurosci. 2020;40(6):1311–1320. 31852732 10.1523/JNEUROSCI.1485-19.2019 PMC7002153

[27] Heim S , Eickhoff SB, Amunts K. Specialisation in broca's region for semantic, phonological, and syntactic fluency? Neuroimage. 2008;40(3):1362–1368. 18296070 10.1016/j.neuroimage.2008.01.009

[28] Gesierich B , Jovicich J, Riello M, et al Distinct neural substrates for semantic knowledge and naming in the temporoparietal network. Cereb Cortex. 2011;22(10):2217–2226. 22047967 10.1093/cercor/bhr286 PMC3895951

[29] Mandelli ML , Welch AE, Vilaplana E, et al Altered topology of the functional speech production network in non-fluent/agrammatic variant of PPA. Cortex. 2018;108:252–264. 30292076 10.1016/j.cortex.2018.08.002 PMC6317366

[30] Slegers A , Filiou R-P, Montembeault M, Brambati SM. Connected speech features from picture description in Alzheimer's disease: A systematic review. J Alzheimer's Dis. 2018;65 (2):519–542. 30103314 10.3233/JAD-170881

[31] Vogel AP , Poole ML, Pemberton H, et al Motor speech signature of behavioral variant frontotemporal dementia: Refining the phenotype. Neurology. 2017;89(8):837–844. 28733335 10.1212/WNL.0000000000004248

[32] Utianski RL , Botha H, Duffy JR, et al Rapid rate on quasi-speech tasks in the semantic variant of primary progressive aphasia: A non-motor phenomenon? J Acoust Soc Am. 2018;144(6):3364–3364. 30599666 10.1121/1.5082210 PMC6296908

[33] Mesulam MM , Wieneke C, Thompson C, Rogalski E, Weintraub S. Quantitative classification of primary progressive aphasia at early and mild impairment stages. Brain. 2012;135(Pt 5):1537–1553. 22525158 10.1093/brain/aws080 PMC3577099

[34] Thompson CK , Cho S, Hsu CJ, et al Dissociations between fluency and agrammatism in primary progressive aphasia. Aphasiology. 2012;26(1):20–43. 22199417 10.1080/02687038.2011.584691 PMC3244141

[35] Meteyard L , Patterson K. The relation between content and structure in language production: An analysis of speech errors in semantic dementia. Brain Lang. 2009;110(3):121–134. 19477502 10.1016/j.bandl.2009.03.007

[36] Themistocleous C , Webster K, Afthinos A, Tsapkini K. Part of speech production in patients with primary progressive aphasia: An analysis based on natural language processing. Am J Speech Lang Pathol. 2021;30(1S):466–480. 32697669 10.1044/2020_AJSLP-19-00114 PMC8702871

[37] Taylor-Rubin C , Croot K, Power E, Savage SA, Hodges JR, Togher L. Communication behaviors associated with successful conversation in semantic variant primary progressive aphasia. Int Psychogeriatr. 2017;29(10):1619–1632. 28593829 10.1017/S1041610217000813

[38] Gola KA , Thorne A, Veldhuisen LD, et al Neural substrates of spontaneous narrative production in focal neurodegenerative disease. Neuropsychologia. 2015;79(Pt A):158–171. 26485159 10.1016/j.neuropsychologia.2015.10.022 PMC4809527

[39] Knibb JA , Woollams AM, Hodges JR, Patterson K. Making sense of progressive non-fluent aphasia: An analysis of conversational speech. Brain. 2009;132(Pt 10):2734–2746. 19696033 10.1093/brain/awp207 PMC2766235

[40] Taylor C , Croot K, Power E, Savage SA, Hodges JR, Togher L. Trouble and repair during conversations of people with primary progressive aphasia. Aphasiology. 2014;28(8–9):1069–1091.

[41] Pressman PS , Simpson M, Gola K, et al Observing conversational laughter in frontotemporal dementia. J Neurol Neurosurg Psychiatry. 2017;88(5):418–424. 28235777 10.1136/jnnp-2016-314931 PMC5726511

[42] Pineault J , Jolicœur P, Grimault S, et al A MEG study of the neural substrates of semantic processing in semantic variant primary progressive aphasia. Neurocase. 2019;25(3-4):118–129. 31256711 10.1080/13554794.2019.1631853

[43] Borghesani V , Dale CL, Lukic S, et al Neural dynamics of semantic categorization in semantic variant of primary progressive aphasia. eLife. 2021;10:e63905. 34155973 10.7554/eLife.63905 PMC8241439

[44] Viard A , Desgranges B, Matuszewski V, et al Autobiographical memory in semantic dementia: New insights from two patients using fMRI. Neuropsychologia. 2013;51(13):2620–2632. 23954715 10.1016/j.neuropsychologia.2013.08.007

[45] Viard A , Piolino P, Belliard S, de La Sayette V, Desgranges B, Eustache F. Episodic future thinking in semantic dementia: A cognitive and fMRI study. PLoS One. 2014;9(10):e111046. 25333997 10.1371/journal.pone.0111046 PMC4205092

[46] Borghesani V , Hinkley LBN, Ranasinghe KG, et al Taking the sublexical route: Brain dynamics of reading in the semantic variant of primary progressive aphasia. Brain. 2020;143(8):2545–2560. 32789455 10.1093/brain/awaa212 PMC7447517

[47] Mummery CJ , Patterson K, Wise RJS, Vandenbergh R, Price CJ, Hodges JR. Disrupted temporal lobe connections in semantic dementia. Brain. 1999;122(1):61–73. 10050895 10.1093/brain/122.1.61

[48] Wilson SM , Brambati SM, Henry RG, et al The neural basis of surface dyslexia in semantic dementia. Brain. 2008;132(1):71–86. 19022856 10.1093/brain/awn300 PMC2638692

[49] Popal H , Quimby M, Hochberg D, Dickerson BC, Collins JA. Altered functional connectivity of cortical networks in semantic variant primary progressive aphasia. NeuroImage Clin. 2020;28:102494. 33395985 10.1016/j.nicl.2020.102494 PMC7708956

[50] Zhou J , Greicius MD, Gennatas ED, et al Divergent network connectivity changes in behavioural variant frontotemporal dementia and Alzheimer's disease. Brain. 2010;133(5):1352–1367. 20410145 10.1093/brain/awq075 PMC2912696

[51] Fredericks CA , Sturm VE, Brown JA, et al Early affective changes and increased connectivity in preclinical Alzheimer's disease. Alzheimer's Dementia. 2018;10:471–479. 10.1016/j.dadm.2018.06.002 PMC6174255 30302368

[52] Balthazar ML , Pereira FR, Lopes TM, et al Neuropsychiatric symptoms in Alzheimer's disease are related to functional connectivity alterations in the salience network. Hum Brain Mapp. 2014;35(4):1237–1246. 23418130 10.1002/hbm.22248 PMC6868965

[53] Stefaniak JD , Alyahya RSW, Lambon Ralph MA. Language networks in aphasia and health: A 1000 participant activation likelihood estimation meta-analysis. Neuroimage. 2021;233:117960. 33744459 10.1016/j.neuroimage.2021.117960

[54] Turkeltaub PE , Messing S, Norise C, Hamilton RH. Are networks for residual language function and recovery consistent across aphasic patients? Neurology. 2011;76(20):1726–1734. 21576689 10.1212/WNL.0b013e31821a44c1 PMC3100133

[55] Pagnoni I , Gobbi E, Premi E, et al Language training for oral and written naming impairment in primary progressive aphasia: A review. Transl Neurodegener. 2021;10(1):24. 34266501 10.1186/s40035-021-00248-z PMC8282407

[56] Volkmer A , Rogalski E, Henry M, et al Speech and language therapy approaches to managing primary progressive aphasia. Pract Neurol. 2020;20(2):154–161. 31358572 10.1136/practneurol-2018-001921 PMC6986989

[57] Henry ML , Hubbard HI, Grasso SM, et al Treatment for word retrieval in semantic and logopenic variants of primary progressive aphasia: Immediate and long-term outcomes. J Speech Lang Hear Res. 2019;62(8):2723–2749. 31390290 10.1044/2018_JSLHR-L-18-0144 PMC6802912

[58] Collins JA , Montal V, Hochberg D, et al Focal temporal pole atrophy and network degeneration in semantic variant primary progressive aphasia. Brain. 2017;140(2):457–471. 28040670 10.1093/brain/aww313 PMC5278308

[59] Seeley WW , Crawford RK, Zhou J, Miller BL, Greicius MD. Neurodegenerative diseases target large-scale human brain networks. Neuron. 2009;62(1):42–52. 19376066 10.1016/j.neuron.2009.03.024 PMC2691647

[60] Carrera E , Tononi G. Diaschisis: Past, present, future. Brain. 2014; 137(Pt 9):2408–2422. 24871646 10.1093/brain/awu101

[61] Hickok G , Poeppel D. Dorsal and ventral streams: A framework for understanding aspects of the functional anatomy of language. Cognition. 2004;92(1-2):67–99. 15037127 10.1016/j.cognition.2003.10.011

