# Supplementary material for: Spared speech fluency is associated with increased functional connectivity in the speech production network in semantic variant primary progressive aphasia
Source: Brain Commun. 2023 Mar 16;5(2):fcad077. doi: 10.1093/braincomms/fcad077 (PMC10082556; doi:10.1093/braincomms/fcad077)
Supplement: fcad077_Supplementary_Data [file fcad077_supplementary_data.pdf]

Supplementary Table 1. Partial correlations between all speech production and lexico-semantic measures, in svPPA patients, controlling for Clinical Dementia Rating scale. (Values shown are pearson r; * p < .05; ** p < .01; *** p < .001).

	Verbal agility task	Speech rate	Articulation rate	PPVT	Average nouns frequency	Average nouns' AoA
**Verbal agility task**	1					
**Speech rate**	.489**	1				
**Articulation rate**	.350	.707***	1			
**PPVT**	.072	.236	.176	1		
**Average nouns frequency**	-.193	.027	.101	-.604**	1	
**Average nouns' AoA**	-.047	-.153	-.154	.434*	-.711***	1

PPVT = Peabody Picture Vocabulary Test; AoA = Age of acquisition

Supplementary table 2. Significant clusters of the speech production and semantic networks in healthy controls and svPPA patients combined.

	Coordinates				
	x	y	z	T value	Cluster size
**Speech production network**					
Left opIFG	-50	8	23	48	45227
Right opIFG	50	10	24	26	*
Left triIFG	-50	30	18	22	*
Right triIFG	48	32	16	20	*
Left SPL	-30	-60	54	14	*
Left SMG	-56	-30	44	18	*
Right SMG	56	-28	44	14	*
SMA	-4	6	54	17	*
Left Intraparietal sulcus	-28	-72	34	11	*
Left MTG	-60	-56	2	14	*
Right Intraparietal sulcus	30	-70	36	11	*
Left Insula	-38	18	6	10	*
Right Insula	32	20	4	10	*
Left Putamen	-34	-6	-4	16	422
Right Putamen	34	-4	-6	12	278
Right MTG	62	-54	-6	9	531
**Semantic network**					
Left aMTG	-62	-6	-18	55	7555
Left Angular gyrus	-56	-60	20	21	*
Right aMTG	62	-2	-16	21	4609
Right Angular gyrus	56	-62	18	15	*
Medial prefrontal cortex	0	50	24	18	6555
Posterior cingulate cortex	0	-60	32	18	4182
Left MFG	-36	8	62	10	556
